# Immunonutritional effects elicited by a novel multicomponent food supplement in children with cow's milk allergy: results from a randomized, placebo-controlled trial

**DOI:** 10.3389/falgy.2026.1760231

**Published:** 2026-03-16

**Authors:** Laura Carucci, Erika Caldaria, Franca Oglio, Raffaele Federico Iorio, Vittoria Mauriello, Antonio Masino, Serena Coppola

**Affiliations:** 1 Department of Translational Medical Science, University Federico II, Naples, Italy; 2 NutriTechLab at CEINGE Advanced Biotechnologies, University Federico II, Naples, Italy

**Keywords:** butyrate, DHA, food allergy, immune tolerance, *L. rhamnosus* GG, malnutrition, Perilla frutescens, Quercitin

## Abstract

**Introduction:**

Cow's milk allergy (CMA) is one of the most common food allergies in childhood frequently associated with body growth impairment and micronutrient deficiencies. Immunonutrition approach with selected bioactive compounds may have beneficial effects on nutritional status and immune tolerance mechanisms. We evaluated the effects of an immunonutrition approach, based on the use of a novel multicomponent food supplement containing prebiotics, postbiotics, vitamin D3, docosahexaenoic acid (DHA), *Perilla frutescens* extracts, and Quercetin in children with CMA.

**Methods:**

Randomized, double-blind, placebo-controlled pilot trial, involving 30 pediatric CMA patients (both sexes, age 36–60 months) randomly assigned to receive the study product or placebo (maltodextrins) for 6 months. The active study product and placebo were provided as powder sachets with identical features. Primary outcomes were changes in body growth. Co-primary exploratory outcomes were serum 25-hydroxyvitamin D (25(OH)D) and DHA levels. Secondary endpoints included the evaluation of Th2 interleukins (ILs) and IL-10, regulatory T cells (Tregs), and growth factors and cytokines modulating ILs production (Tgfb1, Ifna2, Ptgs2, Csf2) in peripheral blood mononuclear cells (PBMCs) collected from CMA pediatric patients.

**Results:**

All participants completed the study without adverse events and with >90% adherence to the allocated treatment. At 6-month follow-up, children in the study product group showed greater improvement in body weight, and height, compared with the patients in the placebo group. Serum 25(OH)D and DHA concentrations significantly improved only in the active study group. In PBMCs collected from the patients, the active study product, but not the placebo exposure, resulted in an inhibition of Th2 cytokines (IL- 4, IL-5, IL-13) response to the stimulation with antigenic peptide β-lactoglobulin and in an increase in IL-10 production and Treg activation rate. The expression of *Tgfb1, Ifna2, Ptgs2, Csf2* resulted also upregulated, suggesting an overall modulation toward immune tolerance in these patients.

**Conclusions:**

This novel multicomponent food supplement improved growth parameters and nutritional status while modulating immune tolerance mechanisms in children with CMA. These findings support the potential of an immunonutrition-based approach using this innovative supplement in managing pediatric food allergy.

**Clinical Trial Registration:**

clinicaltrial.gov, identifier NCT06751810.

## Background

Food allergies (FA) are a growing public health concern, particularly for the pediatric populations living in developed countries. It has been estimated that up to 8% of children globally suffer from FA, and this prevalence has risen substantially over the past two decades (1–5)*.* Food allergies are most common in young children aged <5 years and cow's milk allergy (CMA) is one of the most prevalent FA in early infancy (2, 4).

Food allergies derive from alteration in immune tolerance mechanisms (6–11)*.* Increasing evidence supports the central role of gut microbiome alterations in these processes (12–17). Indeed, alterations in gut microbiome have been linked to enhanced allergens translocation and Th2-skewed immune responses (15–17).

The current standard of care for FA consists of strict elimination of the culprit foods into the diet, however, although effective in preventing acute reactions, this approach carries significant nutritional drawbacks (8). Children on elimination diets are at increased risk of impaired growth and deficiencies in key nutrients, including vitamin D and long-chain *ω*-3 fatty acids, particularly docosahexaenoic acid (DHA) (12–14, 18). Such deficiencies may compromise body growth and immune function (19, 20). Despite the implementation of structured dietary counseling programs, nutritional imbalances remain highly prevalent among FA children (19–22). Several studies have shown that even when counseling is provided by trained dietitians, some deficits persist and are not corrected by standard elimination diets or dietary guidance alone (21, 22). These findings highlight the need for complementary nutritional interventions beyond counseling to support adequate body growth and immune function.

The discovery of the pivotal role of selected dietary factors in influencing the immune system function has introduced the concept of immunonutrition. Several bioactive compounds have been studied for their beneficial modulatory action on immune tolerance mechanisms (23, 34). Among these, increasing evidence supports the use of selected postbiotic products, mainly the gut microbiome-derived metabolite butyrate and the heat-inactivated *L. rhamnosus* GG (LGG) probiotic, in modulating immune tolerance (23–29). Furthermore, specific prebiotic compounds, the Fructooligosaccharides (FOS), have been shown to exert immunomodulatory effects and may help FA treatment by regulating immune system and gut microbiota composition and function (30). Similarly, two plant based compounds, Quercetin and *Perilla frutescens* extracts, also emerged as promising anti-allergic agents (31–34).

The Postbiotics and Plant-derived compounds against Allergy (PPA) project was launched to test the hypothesis that a combined formulation of bioactive compounds could exerts beneficial effects in pediatric patients affected by allergic diseases. Here we provided the results of a clinical trial investigating the effects of a novel multicomponent nutritional supplement containing butyrate, *LGG* postbiotic, FOS, vitamin D, DHA, Quercitin and *Perilla frutescens* extracts on nutritional status and immune tolerance mechanisms in children with CMA.

## Methods

### Study design and study population

This was a single center, randomized, double-blind, placebo-controlled, clinical trial conducted at a tertiary Center for Pediatric Allergy at Federico II University Hospital. The study was designed as a “no-profit” intervention with a duration of six months.

This study included Caucasian pediatric patients aged 36–60 months of both sexes, with IgE-mediated CMA. The CMA diagnosis was based on the presence of clinical features suggestive of CMA, clear response to elimination diet, positive screening test results (skin prick test, serum specific IgE test to cow milk proteins), and/or positive oral food challenge (2, 35).

Exclusion criteria were: non-Caucasian ethnicity, age <36 or >60 months, known hypersensitivity to any of the ingredients of the study product, the presence of other FAs, chronic systemic diseases, immunodeficiencies, treatment with immunomodulators, systemic corticosteroids, antibiotics, or pre-/pro-/syn-/postbiotics within the four weeks prior to enrollment and during the 6-month study period, participation in other studies, and any condition deemed by the investigators to interfere with study participation or outcomes.

### Randomization and intervention

Thirty children fulfilling the inclusion criteria were randomized in a 1:1 ratio to receive daily dietary supplementation with the multicomponent nutritional supplement (active study product) or with placebo for a 6-month period. Randomization was computer-generated, with allocation concealed using sequentially numbered, opaque, sealed envelopes. Investigators, caregivers, and outcome assessors were blinded to treatment allocation throughout the study.

Participants received one sachet daily of the active study product or placebo (composed by maltodextrins, matched for appearance, taste, and smell, without nutritional activity). The composition of the active study product is reported in Table 1. The active study product and the placebo were provided as a powder by AMP Biotech (Benevento, Italy). All components of the study product were food grade. Both active study product and placebo were administered orally mixed with water or foods.

**Table 1:** Composition of the study product.

Ingredients	mg per sachet
Sodium butyrate 98% micro granulated	300
FOS (Fructooligosaccharides)	300
Vitamin D3	0.015
Heat-inactivated *L. rhamnosus* GG (LGG)	300
DHA powder	100
*Perilla frutescens* dry extract (standardized to 2.5% polyphenols)	50
Quercetin 98%	20
Flavors	*q.s.*
Citric acid	*q.s.*
Sucralose	*q.s.*
Isomalt	*q.s.*
TOTAL	2,000

q.s, quantum sufficit.

The study products were provided in consecutively numbered 2 g sachets and stored at room temperature in a dry environment. Only a single batch of both study product and placebo was used throughout the trial, ensuring homogeneity. Products were dispensed to the parents/tutors of each study subject monthly, together with detailed instructions for administration and adherence monitoring. Study adherence was evaluated monthly by counting and weighing the returned sachets and by reviewing the notes recorded in parental diaries. Compliance was considered acceptable with an intake of ≥80% of the recommended treatment.

### Clinical assessments

At baseline (T0), after obtaining written informed consent from the parents or tutors of each subject, the clinical status of the patients was carefully assessed by a multidisciplinary team composed of pediatricians, pediatric allergists, pediatric nurses, and dietitians to exclude those with concomitant comorbidities. Infectious diseases or other conditions were ruled out through a comprehensive anamnestic and physical examination. At enrolment, anamnestic, demographic, anthropometric, and clinical data (including data related to CMA), as well as information on sociodemographic factors, were obtained from the parents/tutors of each patient, collected in a specific clinical chart, and then entered into the study database. All patients received a personalized nutritional counseling by certified pediatric dietitians experienced in the food allergy field, as previously described (21). Briefly, the nutritional counseling was focused on the prescription of a strict cow's milk protein elimination diet, aimed at preventing allergic reactions due to accidental exposure to cow's milk while also avoiding nutritional deficiencies and ensuring optimal growth. Parents/tutors were instructed to exclude cow's milk, dairy products, and foods containing milk from the diet, and were provided with detailed guidance on food label reading, potential cross-reactive foods, and the prevention of cross-contamination. Parents/tutors were provided with detailed instructions for study products administration and adherence monitoring. A follow-up clinical visit was scheduled after six months of treatment (T6). Anthropometric measurements were collected at T0 and T6 following standardized procedures. Briefly, naked subjects were weighed twice on a calibrated electronic scale. Height was measured twice using a standard measuring board. To measure head circumference (the largest occipitofrontal circumference), a non-stretchable measuring tape was used in duplicate. If the anthropometric measurements differed substantially (>100 g for weight and >5 mm for length or head circumference), a third measurement was obtained. Weight-for-age *z*-score (WAZ), height-for-age *z*-score (HAZ), and head circumference-for-age (HCZ) *z*-scores were calculated based upon the World Health Organization (WHO) child growth standards using the WHO Anthro Software (Available at http://www.who.int/childgrowth/software/en/) (36).

In addition, peripheral venous blood samples were collected from all participants at T0 and at T6 for the evaluation of 25(OH)D and DHA serum levels. Moreover, in a representative subgroup of 10 children, an additional volume of blood sample was collected at T0 for the immunological assays on peripheral blood mononuclear cells (PBMCs). Unscheduled visits were made if necessary. Whenever allergic symptoms or other comorbidities occurred, parents were instructed to contact the Center. Adverse events, serious and non-serious, during the 6-month study period were notified by the parents/tutors and coded by diagnosis, severity, date of onset, and resolution by the investigators. They were reported and classified as related (definitely, probably, or possibly related) or unrelated (unlikely or not related) to study products intake, according to the investigators' judgment. All data were collected in the specific clinical chart.

### Laboratory assessment

Serum 25(OH)D levels were quantified by liquid chromatography–tandem mass spectrometry (LC-MS/MS). A 25(OH)D deficiency was defined by the presence of serum level <20 ng/mL, as suggested by the European Society for Pediatric Gastroenterology Hepatology and Nutrition (37).

DHA levels were determined by gas chromatography coupled with flame ionization detection (GC-FID) and expressed as a percentage of weight/weight of total fatty acids. All blood samples were collected in the morning after an overnight fast.

The PBMCs from 10 patients were isolated from heparinized venous blood by density-gradient centrifugation on Ficoll-Paque PLUS (GE Healthcare). The PBMC layer was collected, washed twice with phosphate-buffered saline (PBS), and resuspended in RPMI-1640 medium supplemented with 10% fetal bovine serum (FBS), 1% non-essential amino acids, and 1% penicillin-streptomycin.

Cells were plated at a density of 1 × 10^6^ cells/mL and stimulated with β-lactoglobulin (BLG, 200 µg/mL) in the presence of absence of study product (10 µg/mL) for two days. Non-stimulated PBMCs served as negative controls.

The ILs production was measured in cells culture supernatant. Th2 ILs (IL-4, IL-5, IL-13) and IL-10 were quantified using commercial ELISA kits (R&D Systems, Minneapolis, MN, USA), according to the manufacturer's instructions. The minimum detection concentrations were 31.25 pg/mL for IL- 4, 15.6 pg/mL for IL-5 and IL-13, and 1.6 pg/mL for IL-10.

Flow cytometry was used to determine the frequency of regulatory T cells (Tregs), defined as CD4^+^CD25^+^FoxP3^+^ cells, with fluorochrome-conjugated monoclonal antibodies (BD Biosciences, San Jose, CA, USA). Specific monoclonal antibodies (anti-human CD4, anti-human CD25 and anti-human FoxP3; Cytosens) were used. A total of 50,000 events were acquired for analysis, after gating lymphocytes based on the FSC/SSC dot plot. Data were acquired on a FACS Calibur cytometer (BD Biosciences) and analyzed with FlowJo software.

For gene expression analysis, total RNA was extracted from PBMCs using the RNeasy Mini Kit (Qiagen, Hilden, Germany) quantified using the NanoDrop 2000c spectrophotometer (Thermo Scientific) and purity was verified by A260/280 and A260/230 absorbance ratios. Complementary DNA (cDNA) was synthesized with the High-Capacity cDNA Reverse Transcription Kit (Applied Biosystems, Foster City, CA, USA) according to the manufacturer's instructions and stored at −80 °C until use. Quantitative real-time PCR (qPCR) with SYBR Green chemistry (Applied Biosystems) was employed to assess the expression of the expression of key genes associated with the immune tolerance mechanisms (*Tgfb1*, *Ifna2*, *Ptgs2*, *Csf2*). The amplification protocol was 40 cycles of 15 s for denaturation at 95 °C, 60 s of annealing at 60 °C, and 60 s of elongation at 60 °C in a Light Cycler 7900HT (Applied Biosystem, Grand Island, NY, USA). Relative gene expression was calculated using the ΔΔCt method, normalized to GAPDH (38).

### Data entry

Data were recorded anonymously in case report forms (CRFs). Completeness and accuracy were verified by two researchers. Data were entered into a secure database and reviewed by a biostatistician for data cleaning and analysis before database locking.

### Study outcomes

The primary outcome was the change in growth parameters, expressed as body weight, body height and head circumference, assessed at T0 and T6.

A co-primary exploratory outcome was the change from T0 to T6 in 25(OH)D and DHA serum levels.

Secondary outcomes were:

- the assessment of immune tolerance mechanisms in PBMCs, specifically Th2 (IL-4, IL-5, IL-13) cytokines and IL-10 production, the rate of activated regulatory T cells (CD4^+^CD25^+^FoxP3^+^), and the expression of growth factors and cytokines modulating ILs production (*Tgfb1*, *Ifna2*, *Ptgs2*, *Csf2*);
- compliance with the study products, assessed by caregiver diaries and sachet counts;
- safety and tolerability, evaluated through the incidence of adverse events (e.g., gastrointestinal symptoms, infections, or other intercurrent conditions) monitored throughout the study.

### Sample size

This was conceived as a pilot RCT. No formal sample size calculation was performed. A total of 30 children (15 per group) were enrolled based on feasibility considerations and the exploratory nature of the study. The sample was considered sufficient to provide preliminary estimates of clinical and immunological outcomes, and to inform the design of adequately powered future multicenter trials (39, 40)*.*

### Statistical analysis

Normality of distribution was assessed with the Kolmogorov–Smirnov test. Continuous variables with normal distribution were reported as mean ± standard deviation (SD), while skewed data were expressed as median and interquartile range (IQR). Categorical variables were presented as absolute numbers and percentages.

Comparisons between baseline and follow-up within groups were performed using paired Student's *t*-test or Wilcoxon signed-rank test, as appropriate. Differences between groups (intervention vs. placebo) were evaluated using unpaired Student's *t*-test or Mann–Whitney *U* test. For categorical variables, *χ*² or Fisher's exact test was applied. A two-sided *p*-value <0.05 was considered statistically significant. In addition, correlation analyses were performed to explore associations between changes in biochemical parameters (Δ serum 25(OH)D and Δ DHA) and changes in anthropometric measures (Δ body weight and Δ body height) between baseline (T0) and 6 months (T6). Pearson's correlation coefficient was used to assess linear relationships.

Statistical analyses were performed using SPSS software version 27.0 (IBM Corp., Armonk, NY, USA) and GraphPad Prism version 10 (GraphPad Software, La Jolla, CA, USA).

## Results

### Study population

**Figure 1:**
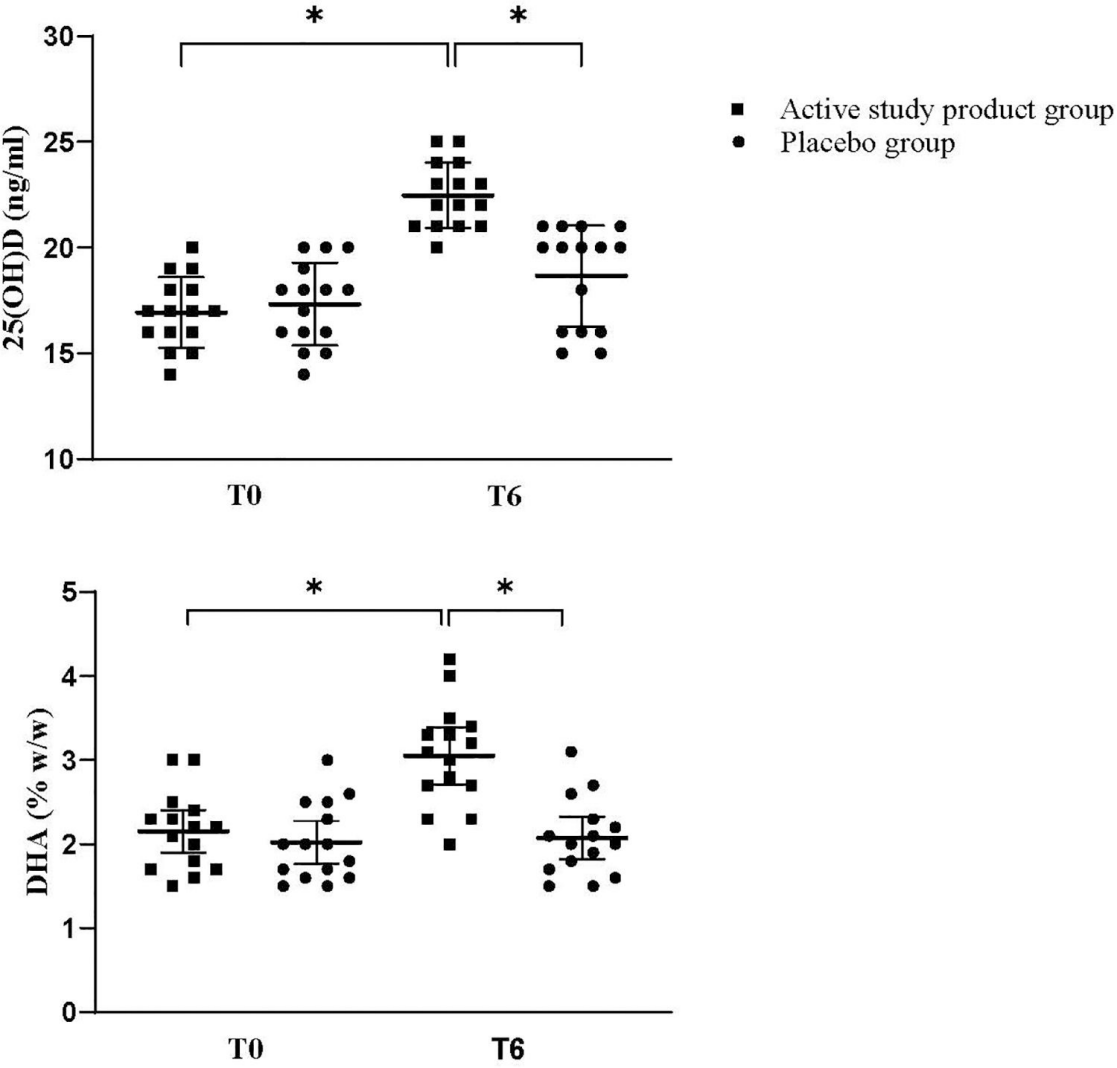
Serum levels of 25-hydroxyvitamin D [25(OH)D] and docosahexaenoic acid (DHA) at baseline (T0) and at 6 months (T6) in the study groups. *p < 0.05. Mean and 95%CI.

A total of 30 children with IgE-mediated CMA were enrolled and randomized to receive either the active study product (*n* = 15) or the placebo (*n* = 15) for 6 months. The study started in December 2024 and ended in June 2025. All participants completed the study, and no protocol deviations were reported. Baseline demographic and clinical characteristics, including age, sex distribution, and serum 25(OH)D and DHA levels, were comparable between the two study groups (Table 2 and Figure 1).

**Table 2:** Demographic and anamnestic features of subjects at enrolment.

Features	Active study product group *N* = 15	Placebo group *N* = 15
Male	12 (80%)	12 (80%)
Cesarean delivery	10 (66.7%)	8 (53.3%)
Born at term	13 (86.7%)	11 (73.3%)
Breastfed for at least 2 months	6 (40%)	10 (66.7%)
Weaning (months)	5 (2)	5 (2)
Familial risk of allergy	12 (80%)	7 (46.7%)
Age at enrollment, months (mean, SD)	41.9 (3.7)	41.6 (3.8)
Positive prick by prick test for fresh milk	15 (100%)	15 (100%)
Gastrointestinal symptoms at CMA onset	8 (53.3%)	11 (73.3%)
Cutaneous symptoms at CMA onset	9 (60%)	5 (33.3%)
Respiratory symptoms at CMA onset	2 (13.3%)	4 (26.7%)

CMA, cow milk allergy. Continuous variables are reported as 50th (median), and interquartile range (IQR) when not specified. Discrete variables are reported as the number and proportion of subjects with the characteristic of interest.

### Primary outcomes

**Figure 2:**
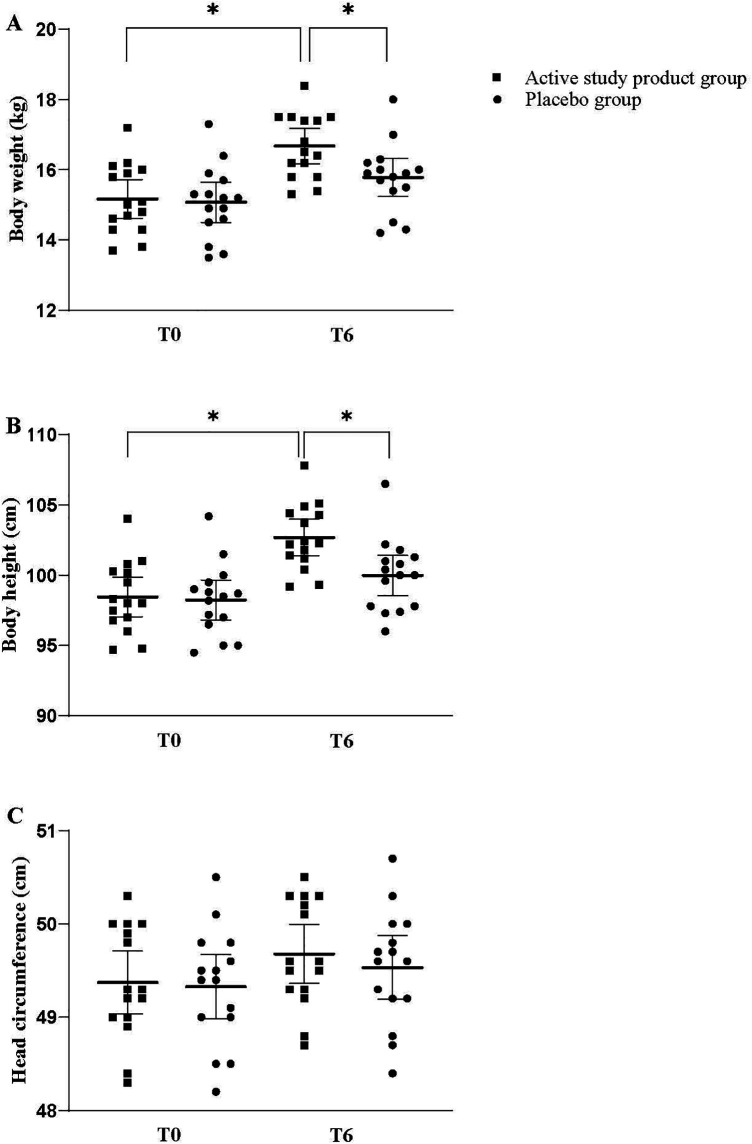
Body weight **(A)**, body height **(B)**, and head circumference **(C)** at baseline (T0) and at 6 months (T6) in the study groups. *p < 0.05, Mean and 95%CI.

**Figure 3:**
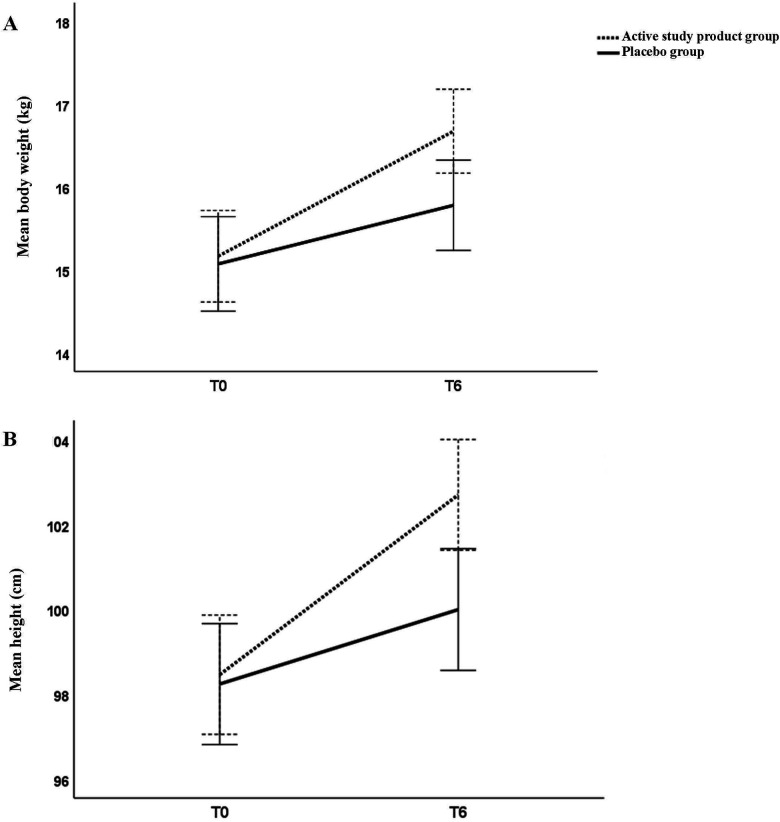
Line charts showing mean (95% CI) changes in body weight **(A)** and body height **(B)** between baseline (T0) and 6 months (T6) in the study groups. *p < 0.05. Mean and 95%CI.

Children supplemented with the active study product exhibited a higher improvement of body growth compared with those receiving placebo after 6-month treatment. At T6, a significant increase in body weight and body height, was observed in the intervention but not in placebo group (Figure 2, panels A,B). Head circumference showed a modest but not significant gain (Figure 2, panel C). Similarly, in the study product group, mean WAZ and HAZ progressively increased during the study period, reaching statistically significant differences at T6 compared with the placebo group. At T6, mean WAZ was 0.19 (95% CI: −0.11 to 0.48) in the active study group vs. −0.23 (95% CI: −0.35 to −0.11) in the placebo group (*p* < 0.05). Similarly, mean HAZ at T6 was −0.27 (95% CI: −0.70 to 0.17) in the active study group compared with −0.88 (95% CI: −1.00 to −0.75) in the placebo group. To provide a clearer visualization of growth trajectories over time, line charts illustrating changes in body weight and body height between baseline (T0) and 6 months (T6) for both active study product and placebo groups are provided in Figure 3. The Figure 1 shows that at T0, both groups had comparable mean (±SD) serum values of 25(OH)D and that after 6 months of dietary intervention, mean serum 25(OH)D levels significantly increased in the intervention group, but not in the placebo group. Finally, although similar baseline DHA concentration into the two study groups, only the active study product group showed a significant increase in DHA levels after 6-month treatment (Figure 1).

Correlation analyses were performed to explore the association between changes in body growth outcomes and biochemical parameters from T0 to T6. Changes in body weight and body height were strongly correlated (*r* = 0.947, *p* < 0.001). Changes in serum 25(OH)D and DHA levels showed a strong positive correlation (*r* = 0.727, *p* < 0.001), reflecting treatment adherence. A positive trend was observed between changes in serum 25(OH)D and body height (*r* = 0.360, *p* = 0.051) and between changes in DHA levels and body height (*r* = 0.349, *p* = 0.058), although these did not reach conventional statistical significance. No significant correlations were observed between changes in biochemical parameters and body weight.

### Secondary outcomes—direct immunomodulatory actions on human PBMCs

To explore the hypothesis that the novel food supplement could modulate immune tolerance mechanisms in CMA patients, PBMCs were isolated from a representative subgroup of 10 children at T0 before treatment. Cells were stimulated *in vitro* with BLG in the presence or absence of the active study product.

**Figure 4:**
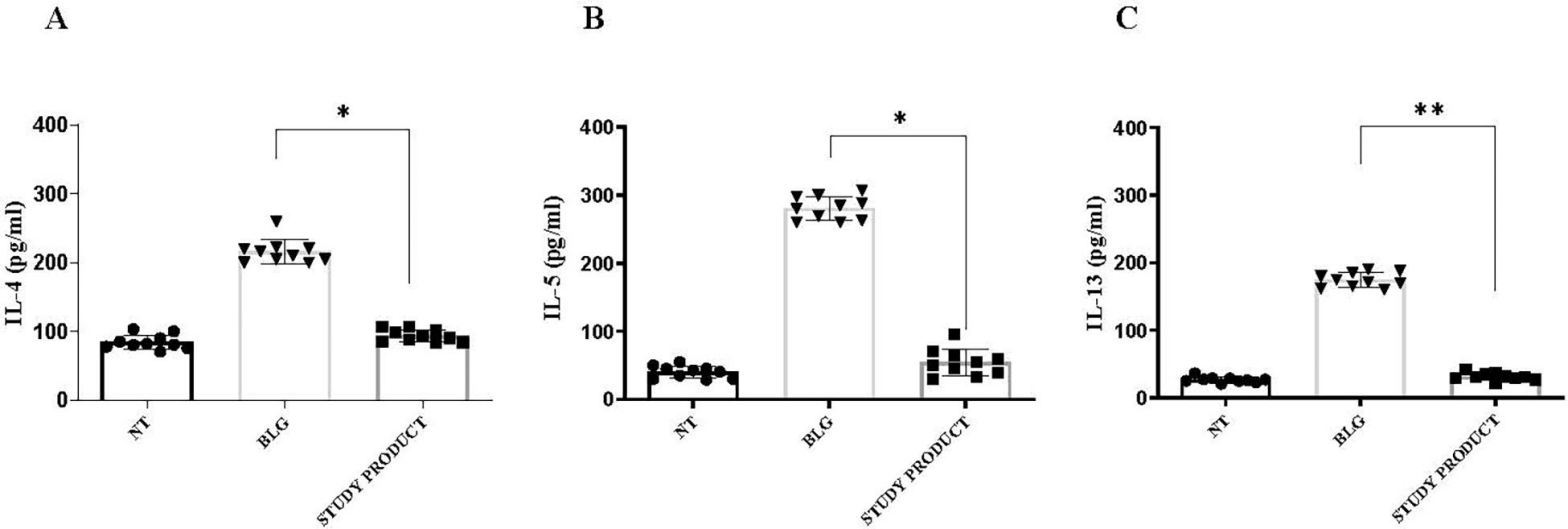
Effect of the study product on the production of Th2 cytokines in PBMCs collected from children with cow's milk protein allergy. In response to β-lactoglobulin stimulation, significant increases in IL-4 **(A)**, IL-5 **(B)**, and IL-13 **(C)** were observed. The concomitant treatment with study product significantly reduced the production of these pro-inflammatory cytokines. Data are expressed as mean ± SD. *p < 0.05; **p < 0.01. NT, medium; BLG, β-lactoglobulin.

**Figure 5:**
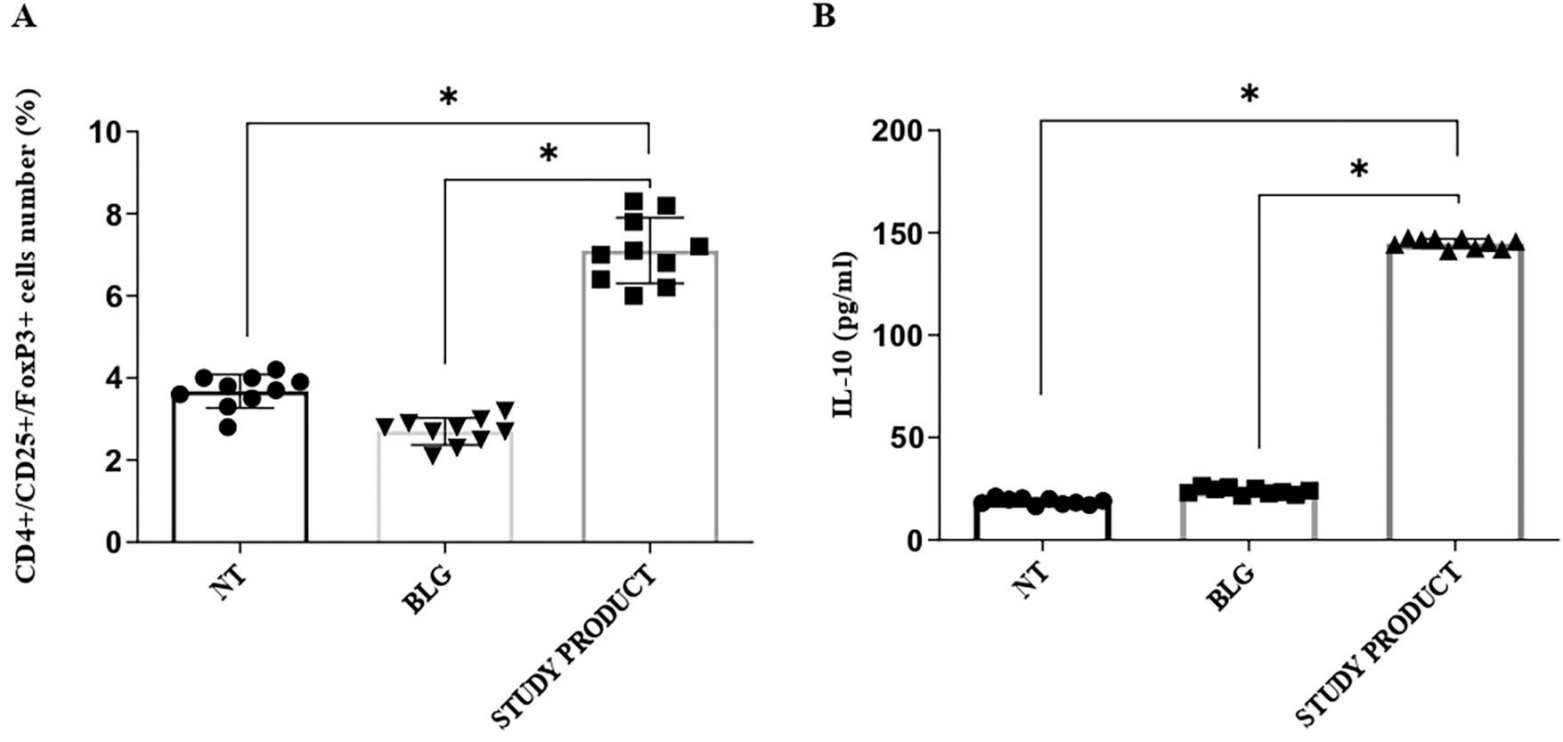
Effect of the study product on the activated regulatory T cells (tregs) rate and IL-10 production in PBMCs from children with cow's milk protein allergy. Study product treatment, in combination with β-lactoglobulin stimulation, significantly increased the proportion of CD4^+^CD25^+^FoxP3^+^ Tregs **(A)** and enhanced IL-10 secretion **(B)** *p < 0.05. NT, medium; BLG, β-lactoglobulin.

**Figure 6:**
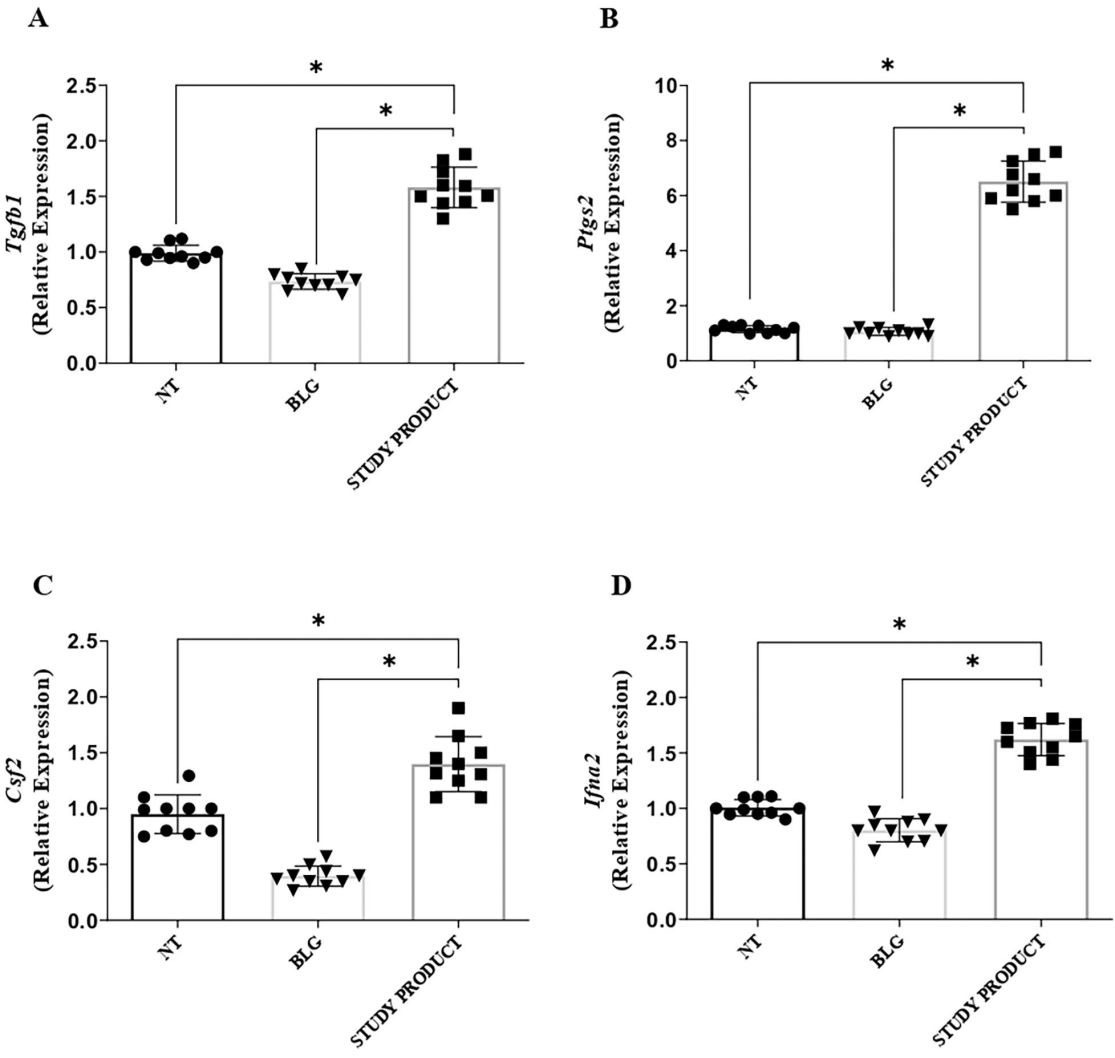
Effect of the study product on the activation of regulatory growth factors and cytokines modulating interleukins production in PBMCs from children with cow's milk protein allergy. Gene expression of *Tgfb1* **(A)**, *Ptgs2* **(B)**, *Csf2* **(C)**, and *Ifna2* **(D)** was significantly upregulated in PBMCs exposed to β-lactoglobulin plus study product compared with β-lactoglobulin alone, indicating that the supplement drives immune cells toward a regulatory phenotype. *p < 0.05. NT, medium; BLG, β-lactoglobulin.

As expected, the PBMCs stimulated with BLG produced elevated levels of Th2 ILs (IL-4, IL-5, IL-13), consistent with the allergic phenotype of the enrolled children. The exposure to the active study product significantly reduced the production of these Th2 cytokines in response to BLG, resulting in levels comparable to those observed in cells not exposed to the food antigen (Figure 4). Conversely, the production of the tolerogenic IL-10 cytokine resulted enhanced in PBMCs exposed to the active study product (Figure 5, panel B, *p* < 0.05). Flow cytometry analysis showed that the food supplement exposure resulted in an increased rate of activated CD4^+^CD25^+^FoxP3^+^ regulatory T cells (Figure 5, panel A, *p* < 0.05). Finally, the expression of the regulatory growth factors and cytokines *Tgfb1*, *Ptgs2*, *Csf2*, and *Ifna2* genes resulted increased after stimulation with the active study product (Figure 6). Altogether these results suggest that the novel multicomponent food supplement can elicit an immunomodulatory action toward immune tolerance.

### Safety and tolerability

The active study product and the placebo were well tolerated by the study patients. No adverse events were registered during the study. Compliance, assessed through parental diaries and sachets count, exceeded 90% in both groups, confirming good acceptability of the intervention.

## Discussion

The results obtained in this randomized, double-blind, placebo-controlled trial provide evidence supporting the efficacy of the immunonutritional approach based on the use of a novel multicomponent food supplement in pediatric patients affected by FA. The results suggest that this immunonutritional approach could be able not only to improve body growth and to correct key micronutrient deficiencies, but also to modulate immune tolerance mechanisms.

The improvement in anthropometric parameters observed in the trial highlights the beneficial nutritional impact of the food supplement in FA children on elimination diet, who are particularly vulnerable to growth impairment and micronutrients deficiencies (19–22). Previous studies have shown that children with CMA frequently present impaired linear growth and suboptimal nutrients intake when compared with their healthy peers (41). Similarly, clinical trials in children with CMA confirmed suboptimal growth, underlining the nutritional vulnerability of these patients (42). By providing essential nutrients often lacking in restricted diets—such as vitamin D and DHA—this food supplement supported adequate body growth while ensuring that weight-for-age and height-for-age *z*-scores followed healthier trajectories.

Vitamin D status significantly improved in children receiving the novel food supplement. Deficiency rates dropped dramatically after supplementation, in line with the inclusion of vitamin D3 in the formulation. Beyond its bone health benefits, vitamin D plays a pivotal role in immune regulation, promoting Treg differentiation and inhibiting Th2 response (43, 44)*.* The correction of vitamin D deficiency therefore represents not only a nutritional but also an immunological advantage, potentially contributing to the tolerogenic effects observed at the cellular level.

Similarly, the nutritional intervention with the novel multicomponent food supplement effectively increased DHA serum levels in the intervention group, which may help in downregulating inflammation and in promoting immune tolerance. DHA is an essential long- chain *ω*-3 fatty acid with anti-inflammatory and immunomodulatory properties. Reduced dietary intake of *ω*-3 fatty acids has been associated with a higher risk of allergic diseases (12–14, 45–49)*.*

The anti-inflammatory potential of the novel food supplement ingredients can also be involved in the beneficial impact on body growth observed in this trial. It has been demonstrated that inflammation influence negatively body growth affecting metabolism, bone growth, and the immune response (50–53). Thus, downregulating inflammation in FA patients with this novel food supplement could help the normalization of the body growth pattern.

The PBMCs investigations provided further insights in the anti-inflammatory and tolerogenic mechanisms elicited by the novel food supplement. The exposure to the multicomponent food supplement resulted in a reduction in pro-inflammatory Th2 cytokines production. The effect paralleled with an increased production of the anti-inflammatory cytokine IL-10. Flow cytometry study showed an increased rate of activated CD4^+^CD25^+^FoxP3^+^ Tregs in PBMCs exposed to the multicomponent food supplement, which are pivotal cells for inducing and maintaining immune tolerance (54). Gene expression analyses revealed upregulation of tolerogenic mediators, including *Tgfb1*, *Ifna2*, *Ptgs2*, and *Csf2*. These molecules are known to drive immune cells toward a regulatory phenotype, thereby promoting Treg expansion and suppressing inflammatory Th2-oriented response (44, 54).

A major limitation of this study is that we did not explore which component of the novel food supplement could be responsible for each effect observed in the trial. It is possible to speculate that all components of the novel food supplement could be involved in these immunoregulatory actions. It has been demonstrated that FOSs can positively influence dendritic cells, which can increase IgA secretion and Treg production in FA (55)*.* Evidence from both human studies (24) and animal models (56) highlighted the potential of butyrate as a protective gut microbiome-derived metabolite capable to promote potent anti-inflammatory and anti-allergic effects and to promote immune tolerance, also through an epigenetic modulation of gene expression (24, 56–59). Similarly, recent evidence suggest that the heat-inactivated LGG postbiotic adds an additional layer of immune support, regulating autophagy, Tregs activation, cytokines production, and epithelial gut barrier integrity (25–27, 60).

It is also important that collectively FOS, butyrate, and LGG postbiotic can exert a beneficial modulation of gut microbiome structure and function, increasing the abundance of microorganisms with protective actions against allergic inflammation (25–27, 57–60). Another major limitation of the present study is that we didn't evaluate the impact of the novel food supplement on gut microbiome of children affected by FA. These effects should be assessed in future studies involving metagenomic and metabolomic investigations.

The novel food supplement contains two plant-derived bioactive compounds, Quercetin and *Perilla frutescens* extracts. Quercetin is among the most thoroughly investigated dietary flavonoids, recognized for its anti-allergic activity by suppressing mast cell degranulation, and reducing pro-inflammatory signalling. Additionally, it can shape gut microbiota toward an increased abundance of SCFA-producing bacterial taxa (61, 62).

*Perilla frutescens*, an edible herb traditionally used in Asian cuisine characterized by a high polyphenol content, has also been suggested as a functional ingredient against allergy. It exhibits protective effects against oxidative stress and inflammation (63, 64). Both Quercetin and *Perilla frutescens* extracts contribute to the stabilization of epithelial gut barrier function and to the down-regulation of mucosal inflammation through the activation of the NF-E2 p45-related factor 2 (Nrf2) pathway, nuclear factor kappa B (NF-κB) and STAT3 modulation, and the upregulation of genes encoding tight junction proteins (31–34, 65). Notably, the co-administration of Quercetin extracts has been proposed to potentiate the antihistaminic and anti-inflammatory properties of *Perilla frutescens* (63).

Lastly, other limitations of the study are related to the relatively small sample size, the pilot nature of the trial, and short follow-up period that were insufficient to evaluate immune tolerance acquisition in CMA children. The homogeneity of the study population, which included only Caucasian children, reduces the generalizability of the results to broader pediatric populations. Future multicenter randomized controlled trials should aim to include larger, multiethnic cohorts and extend the follow-up to assess immune tolerance outcomes through standardized oral food challenges. These studies should also integrate omics-based tools, including microbiome, metabolomic, and transcriptomic analyses, to better elucidate the mechanistic pathways involved in the observed immunomodulation. Such approaches would provide a comprehensive understanding of how nutritional immunomodulation could complement current allergy management strategies and potentially shift the paradigm toward disease modification.

## Conclusions

In conclusion, the novel multicomponent food supplement while improving body growth and correcting key micronutrient deficiencies in children with CMA, promotes an immunoregulatory action by reducing Th2 responses, enhancing IL-10 secretion and Treg activation, and upregulating tolerogenic dendritic cell markers expression. All together these findings support the potential of this innovative food supplement as an effective immunonutritional approach in patients affected by FA.

## Data Availability

The raw data supporting the conclusions of this article will be made available by the authors, without undue reservation.
